# Chronic alcohol drinking persistently suppresses thalamostriatal excitation of cholinergic neurons to impair cognitive flexibility

**DOI:** 10.1172/JCI154969

**Published:** 2022-02-15

**Authors:** Tengfei Ma, Zhenbo Huang, Xueyi Xie, Yifeng Cheng, Xiaowen Zhuang, Matthew J. Childs, Himanshu Gangal, Xuehua Wang, Laura N. Smith, Rachel J. Smith, Yubin Zhou, Jun Wang

**Affiliations:** 1 Department of Neuroscience and Experimental Therapeutics, College of Medicine, Texas A&M University Health Science Center, Bryan, Texas, USA.; 2 Institute for Neuroscience and; 3 Department of Psychological and Brain Sciences, Texas A&M University, College Station, Texas, USA.; 4 Institute of Biosciences and Technology and; 5 Department of Translational Medical Sciences, College of Medicine, Texas A&M University Health Science Center, Houston, Texas, USA.

**Keywords:** Neuroscience, Addiction, Behavior, Psychiatric diseases

## Abstract

Exposure to addictive substances impairs flexible decision making. Cognitive flexibility is mediated by striatal cholinergic interneurons (CINs). However, how chronic alcohol drinking alters cognitive flexibility through CINs remains unclear. Here, we report that chronic alcohol consumption and withdrawal impaired reversal of instrumental learning. Chronic alcohol consumption and withdrawal also caused a long-lasting (21 days) reduction of excitatory thalamic inputs onto CINs and reduced pause responses of CINs in the dorsomedial striatum (DMS). CINs are known to inhibit glutamatergic transmission in dopamine D1 receptor–expressing medium spiny neurons (D1-MSNs) but facilitate this transmission in D2-MSNs, which may contribute to flexible behavior. We discovered that chronic alcohol drinking impaired CIN-mediated inhibition in D1-MSNs and facilitation in D2-MSNs. Importantly, in vivo optogenetic induction of long-term potentiation of thalamostriatal transmission in DMS CINs rescued alcohol-induced reversal learning deficits. These results demonstrate that chronic alcohol drinking reduces thalamic excitation of DMS CINs, compromising their regulation of glutamatergic transmission in MSNs, which may contribute to alcohol-induced impairment of cognitive flexibility. These findings provide a neural mechanism underlying inflexible drinking in alcohol use disorder.

## Introduction

Alcohol use disorder is a chronic brain disorder characterized by an inability to stop drinking despite the resultant adverse consequences (1, 2). This inability is associated with impaired flexibility in decision making, which contributes to compulsive alcohol use (1–5). Increasing evidence suggests that the dorsomedial striatum (DMS) is involved in cognitive flexibility (6–11). Understanding whether and how chronic alcohol consumption affects striatum-mediated cognitive flexibility will provide therapeutic strategies to treat alcohol use disorder.

In the DMS, cholinergic interneurons (CINs) are the major source of acetylcholine and contribute to cognitive flexibility in response to salient stimuli (12–14). CINs play an essential role in modulating striatal circuit activity, thereby regulating output from the striatum (15–17). The medium spiny neurons (MSNs), which express either dopamine D1 receptors (D1Rs) or D2Rs, are the principal striatal projection neurons. D1R-expressing MSNs (D1-MSNs) and D2-MSNs play different roles in motor control and goal-directed behavior (18–24). Accumulating evidence demonstrates that the characteristic burst-pause firing of CINs regulates MSN activity; this firing pattern is triggered by excitatory inputs from the thalamus, which is a critical modulator of striatal activity (14, 17, 25). MSN regulation by CINs is mediated by the actions of acetylcholine on pre- and postsynaptic muscarinic receptors. For example, burst-associated transient acetylcholine release produces a muscarinic M2/M4 receptor–mediated reduction in glutamate release at corticostriatal terminals on both D1- and D2-MSNs (17, 26). The more prolonged effects of acetylcholine on postsynaptic excitability during the “pause window” are mediated by the preferential activation of muscarinic M1 receptors on D2-MSNs but not D1-MSNs. These studies demonstrated that CIN burst-pause firing following thalamic activation is crucial for the functional modulation of striatal MSNs. Since striatal D1- and D2-MSNs respectively give rise to the direct (“go”) and indirect (“no go”) pathway, CINs stand to allow cognitive flexibility by modulating “go” and “no go” actions (17, 25). Several studies have demonstrated that alcohol preferentially increases glutamatergic transmission in D1-MSNs but not in D2-MSNs, an effect that potentiates the “go” pathway (23, 24, 27–29). However, it remains unclear how alcohol affects CIN-mediated modulation of D1- and D2-MSNs.

In the present study, we demonstrated that chronic alcohol intake and withdrawal impaired cognitive flexibility in reversing action-outcome contingency. We found that chronic alcohol intake reduced thalamic inputs onto CINs. In the meantime, chronic alcohol consumption led to reduced pause responses of CINs along with increased spontaneous firing activities. Moreover, chronic alcohol intake impaired both CIN-mediated inhibition of glutamatergic transmission in D1-MSNs and CIN-mediated short-term facilitation of glutamatergic transmission in D2-MSNs. These results indicate that alcohol consumption is associated with distinctive CIN-mediated changes in different MSN circuits, providing a potential neural mechanism driving the inflexible drinking underlying alcohol use disorder.

## Results

### Chronic alcohol consumption and withdrawal impair reversal of operant learning in rats.

**Figure 1:**
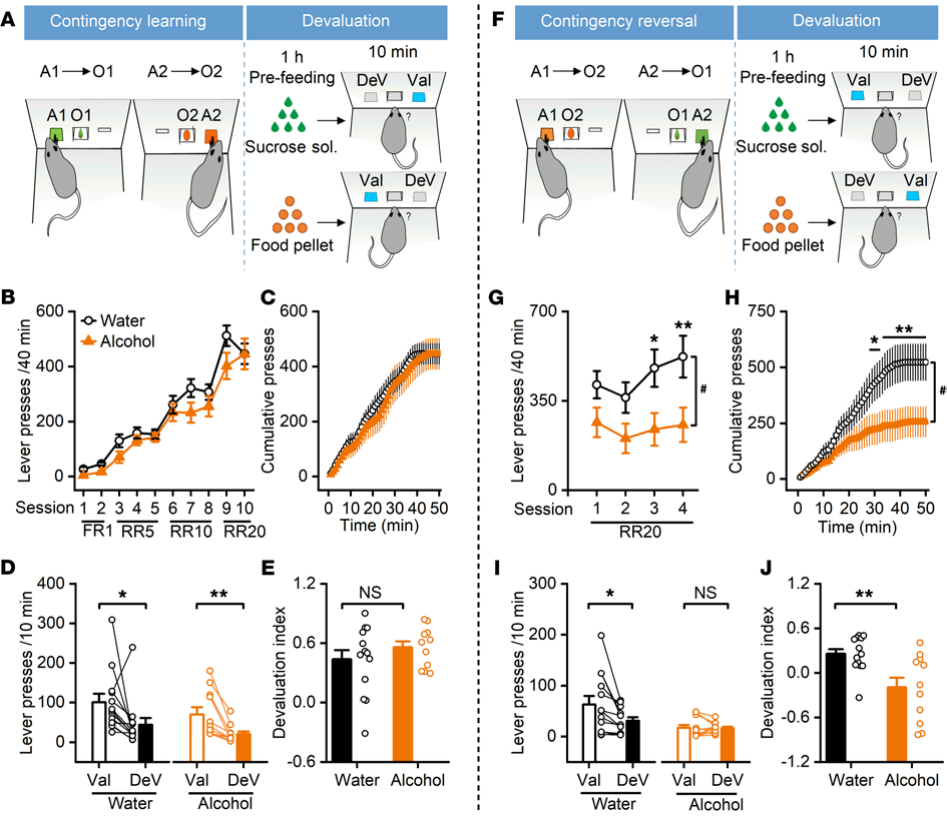
Chronic alcohol intake impairs reversal of instrumental learning. (**A**) Schematic of the instrumental learning procedure and subsequent devaluation testing. Rats consumed 20% alcohol for 8 weeks before operant training. (**B**) The alcohol and water groups did not differ in total lever presses during the acquisition of initial contingencies. (**C**) The alcohol and water groups showed no difference in cumulative lever presses during the last initial training session (session 10). (**D**) Outcome-specific devaluation testing showed that both water and alcohol groups pressed the devalued (DeV) lever significantly fewer times than the valued (Val) lever; *P < 0.05, **P < 0.01. (**E**) The devaluation index, defined as (Val – DeV)/(Val + DeV), did not differ significantly between the 2 groups. (**F**) Schematic of the next round of instrumental learning with reversed contingencies and subsequent devaluation testing. (**G**) The alcohol group showed significantly reduced lever pressing during the reversed contingency training sessions; ^#^*P <* 0.05; *P < 0.05, **P < 0.01. (**H**) The alcohol group showed significantly fewer cumulative lever presses in the last reversal learning session (session 4); ^#^*P* < 0.05; *P < 0.05, **P < 0.01. (**I**) Second devaluation testing showed that the water group interacted less with the DeV lever, but this devaluation was not observed in the alcohol group; *P < 0.05 (Water); NS, *P* > 0.05 (Alcohol). (**J**) The devaluation index was significantly lower in the alcohol group than in the water group; **P < 0.01; *n =* 13 male rats (Water) and 11 male rats (Alcohol) for **B**–**E**, **G**, **I**, and **J**; *n =* 12 male rats (Water) and 10 male rats (Alcohol) for **H**. Two-way repeated-measures (RM) ANOVA (**B**, **C**, **G**, and **H**) followed by Tukey’s post hoc test (**G** and **H**); paired (**D** and **I**) or unpaired (**E** and **J**) *t* test.

Thalamic inputs onto DMS CINs have been implicated in the reversal of instrumental learning (9, 10, 30). We thus examined whether chronic alcohol intake and withdrawal affected the acquisition and reversal of action-outcome contingencies. Rats that had been exposed to water (controls) or 20% alcohol using an intermittent-access 2-bottle choice drinking procedure (24, 31–34) for 8 weeks were trained to learn 2 action-outcome contingencies involving food pellets or sucrose solution (refs. 9, 35, and Figure 1A). The water and alcohol groups both acquired action-outcome contingencies during the increased-effort training schedule (Figure 1B). The total number of lever presses was slightly lower in the alcohol group than in the water group, but this difference was not statistically significant (Figure 1B; *F*_(1,22)_ *=* 3.55, *P* = 0.07). Cumulative lever presses during the last session of the initial learning period did not differ between the 2 groups (Figure 1C; *F*_(1,22)_ = 0.13, *P* > 0.05).

After the initial acquisition of this task, we investigated the sensitivity to outcome devaluation. To achieve this goal, animals were fed with either food pellets or sucrose solution before receiving extinction training, where lever presses were monitored. We found that both alcohol-drinking and water control rats significantly decreased their presses on the outcome-satiated (devalued) lever (Figure 1D; *t*_(12)_ = 2.20, *P* < 0.05 for water group; *t*_(10)_ = 3.71, *P* < 0.01 for alcohol group). Analysis of the devaluation index (the difference between the proportions of nondevalued and devalued lever presses) did not identify any statistically significant difference in the degree of goal-directed versus habitual behavior between the alcohol-drinking and the water control rats (Figure 1E; *t*_(22)_ = –1.02, *P* > 0.05). These results indicated that alcohol-drinking and water control rats showed similar levels of goal-directed behavior.

Next, we examined the flexibility of the rats’ responses to a change in the action-outcome contingency. We reversed the relationship between action and outcome so that pressing the lever previously used to access sucrose solution now led to the delivery of food pellets and vice versa (Figure 1F). Following this contingency reversal, the total lever presses were significantly lower in the alcohol group than in the control group (Figure 1G; *F*_(1,22)_ = 6.28, *P* < 0.05). Cumulative lever presses were also lower in the alcohol-drinking rats than in water controls during the last session of reversal training (Figure 1H; *F*_(1,20)_ = 4.68, *P* < 0.05). These results indicated that chronic alcohol intake and withdrawal (at least 10 days) impaired reversal learning in this task.

Lastly, our analysis of the relative contributions of goal-directed versus habitual behavior following contingency reversal showed that the alcohol group pressed indiscriminately on devalued and nondevalued levers, whereas the water control rats still favored the nondevalued lever (Figure 1I; *t*_(12)_ = 2.87, *P* < 0.05 for water group; *t*_(10)_ = 0.18, *P* > 0.05 for alcohol group). The devaluation index was, therefore, significantly lower in alcohol-drinking rats, as compared with their water controls (Figure 1J; *t*_(22)_ = 3.14, *P* < 0.01). We also compared the difference between the first and second devaluation indices in the 2 study groups; the alcohol group showed a significantly larger decrease than did the water group (*t*_(22)_ = 2.88, *P* < 0.01; Supplemental Figure 1; supplemental material available online with this article; https://doi.org/10.1172/JCI154969DS1). These results indicated that the water controls maintained a goal-directed strategy in response to the new action-outcome association. However, alcohol-drinking rats failed to do so and instead used a strategy more consistent with habitual behavior, suggesting that chronic alcohol intake and withdrawal impaired cognitive flexibility in response to changes in action-outcome associations in rats.

### Chronic alcohol consumption reduces glutamatergic thalamostriatal inputs onto DMS CINs.

**Figure 2:**
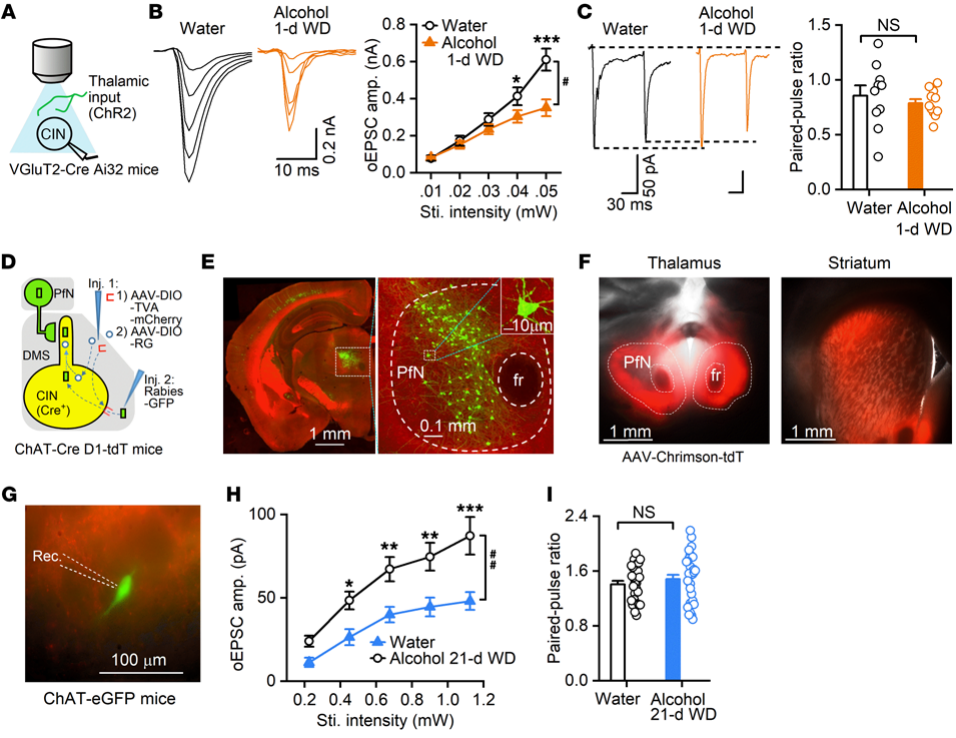
Chronic alcohol consumption reduces thalamostriatal glutamatergic inputs onto DMS CINs. (**A**) Schematic of light stimulation of ChR2-expressing thalamic inputs and whole-cell recording of CINs. (**B**) Chronic alcohol consumption suppressed thalamostriatal inputs onto CINs in DMS slices prepared 24 hours after the last alcohol exposure (1-day withdrawal [WD]); ^#^*P* < 0.05; *P < 0.05, ***P < 0.001; *n =* 11 neurons from 4 male mice (Water) and 13 neurons from 3 male mice (Alcohol). (**C**) Chronic alcohol consumption did not alter the glutamate release probability indicated by paired-pulse measurement; NS, *P* > 0.05; *n =* 10 neurons from 3 male mice (Water) and 11 neurons from 3 male mice (Alcohol). (**D**) Schematic showing viral infusions. We infused helper viruses (AAV-DIO-TVA-mCherry and AAV-DIO-RG) into the DMS of ChAT-Cre D1-tdT mice (Inj. 1) and rabies-GFP into the same site 2 weeks later (Inj. 2). (**E**) Sample coronal images from 4 mice showing that rabies-GFP–labeled PfN neurons projected to DMS CINs. fr, fasciculus retroflexus. (**F**) Sample images of tdT fluorescence in the PfN (injection site) and striatum from 8 mice. (**G**) Sample image from 8 mice showing a GFP-expressing recorded (Rec.) CIN and tdT-positive fiber. (**H**) Input-output curves of oEPSC amplitudes in CINs measured 21 days after the last alcohol exposure (21-d WD) from mice injected with AAV-Chrimson-tdT in the PfN; ^##^*P* < 0.01; *P < 0.05, **P < 0.01, ***P < 0.001; *n =* 15 neurons from 2 male and 2 female mice (Water) and 14 neurons from 4 female mice (Alcohol). (**I**) Paired-pulse ratios in mice injected with AAV-Chrimson-tdT; NS, *P* > 0.05; *n =* 27 neurons from 2 male and 3 female mice (Water) and 32 neurons from 1 male and 4 female mice (Alcohol). Two-way RM ANOVA followed by post hoc test (**B** and **H**); unpaired *t* test (**C** and **I**).

The striatum receives major glutamatergic inputs from both the cortex and thalamus. Reduced flexibility in reversal learning is known to be associated with thalamostriatal transmission in DMS CINs (9, 17, 36). We next investigated whether alcohol consumption altered thalamic inputs onto DMS CINs. To selectively induce thalamostriatal transmission, we expressed channelrhodopsin 2 (ChR2) in thalamic inputs (Figure 2A) by crossing transgenic mice expressing Cre recombinase under the control of the vesicular glutamate transporter 2 (VGluT2) promoter (VGluT2-Cre mice) with transgenic mice expressing Cre-dependent ChR2-eYFP (Ai32 mice) (37). This cross produced VGluT2-Cre Ai32 mice. Previous studies in VGluT2-Cre mice reported that VGluT2-expressing inputs to the striatum mainly arose from the thalamus (38, 39).

CINs are easily distinguished from other striatal cell types because they have a large diameter and unique electrophysiological characteristics (40, 41). We thus distinguished CINs from MSNs by their larger size, spontaneous firing (Supplemental Figure 2A), higher resting membrane potential, characteristic voltage sag in response to hyperpolarizing current injection, and greater excitability in response to depolarizing current injection (Supplemental Figure 2B; resting membrane potentials: *t*_(10)_ = 4.75, *P* < 0.001). Interestingly, repetitive light-mediated stimulation of thalamic inputs in VGluT2-Cre Ai32 mouse slices evoked distinct patterns of excitatory postsynaptic potentials (EPSPs) in CINs and MSNs. We found that the second EPSP was larger than the first EPSP in CINs, while MSNs showed the opposite pattern (Supplemental Figure 2C; *t*_(10)_ = 6.87, *P* < 0.001). We used a combination of these approaches to identify CINs when these neurons did not express fluorescent proteins.

We then explored how chronic alcohol intake influenced thalamostriatal glutamatergic transmission onto DMS CINs. VGluT2-Cre Ai32 mice were trained to consume 20% alcohol for 8 weeks using the intermittent-access 2-bottle choice drinking procedure (23, 33). Twenty-four hours after the last alcohol exposure, we prepared striatal slices and measured optically evoked excitatory postsynaptic currents (oEPSCs) in CINs. We found that the oEPSC amplitude was significantly lower in CINs from the alcohol group than in those from the water control group (Figure 2B; *F*_(1,22)_ = 5.39, *P* < 0.05). This result suggests that chronic alcohol intake reduces thalamostriatal inputs onto DMS CINs. To further investigate the mechanism underlying this reduction, we measured the paired-pulse ratio (PPR) of oEPSCs that were activated by 2 stimuli, delivered 100 milliseconds (ms) apart. This analysis found no significant difference between the alcohol group and the water group (Figure 2C; *t*_(19)_ = 0.72, *P* > 0.05). These results suggested that the reduced thalamostriatal transmission to CINs in mice with chronic alcohol exposure was unlikely to be caused by a reduced probability of presynaptic glutamate release.

To further confirm the alcohol-associated suppression of thalamostriatal transmission, we infused adeno-associated virus (AAV)-Chrimson-tdTomato (tdT) into a thalamic nucleus that is known to project to DMS CINs. Previous studies identified dense inputs to the striatum from multiple thalamic nuclei, including the parafascicular nucleus (PfN) (42, 43). To investigate this, we infused rabies helper viruses into the DMS of ChAT-Cre mice, waited 3 weeks, and then infused rabies-GFP at the same location 3 weeks later (Figure 2D). Two Cre-dependent AAV serotype 8 vectors were employed as helper viruses; one expressed rabies glycoprotein (RG) (AAV8-DIO-RG), and the other expressed an avian membrane EnvA receptor protein (TVA) and mCherry (AAV8-DIO-TVA-mCherry). This approach produced extensive GFP expression in the PfN (Figure 2E), indicating dense innervation of DMS CINs by thalamic PfN neurons. Next, we infused AAV-Chrimson-tdT into the PfN of ChAT-eGFP mice and detected the tdT fluorescent signal in the striatum (Figure 2F). Animals were trained to consume alcohol as described above. Twenty-four hours after the last alcohol exposure, striatal slices were prepared to measure oEPSCs in CINs. Changes in oEPSCs similar to those shown in Figure 2B were observed (Supplemental Figure 3A; *F*_(1,22)_ = 4.74, *P* < 0.05). We did not observe significant changes in PPR measurements (Supplemental Figure 3B; *t*_(39)_ = –1.44, *P* > 0.05). Because we had previously observed behavioral deficits weeks (10-day initial training + 2-day initial devaluation test + 2- to 3-day retraining + 4-day reversal learning + 2-day second devaluation test → 21 days) after stopping alcohol consumption (Figure 1), we also measured oEPSCs 21 days after the last alcohol exposure. Similar results were observed at this time point (oEPSCs, Figure 2H, F_(1,27)_ = 10.91, *P* < 0.01; PPR, Figure 2I, t_(53)_ = –0.72, *P* > 0.05).

Taken together, these data suggest that chronic alcohol consumption causes a long-lasting decrease in thalamostriatal inputs onto DMS CINs.

### Chronic alcohol consumption significantly increases the spontaneous firing of DMS CINs and shortens their pause responses.

**Figure 3:**
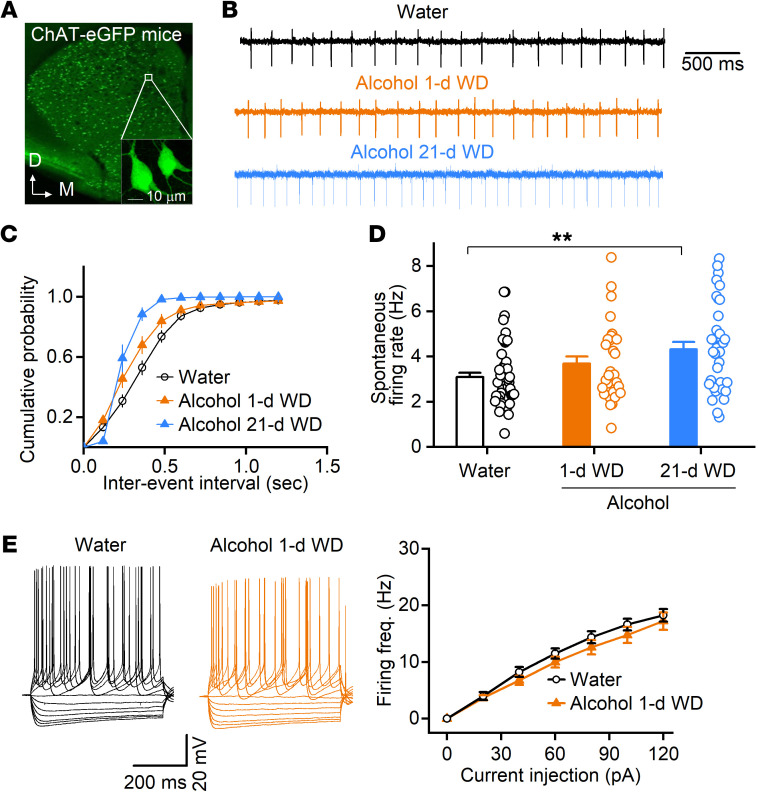
Chronic alcohol consumption increases spontaneous, but not evoked, firing of DMS CINs. ChAT-eGFP mice were trained to consume 20% alcohol for 8 weeks, and DMS slices were prepared 24 hours (1-d WD) and 21 days (21-d WD) after the last alcohol exposure. (**A**) Sample image from 13 mice showing green CINs in the striatum. D, dorsal; M, medial. (**B**) Sample traces of spontaneous CIN firing in the water and alcohol groups using the cell-attached recording. (**C** and **D**) Cumulative plots of the inter-event intervals (**C**) and the spontaneous firing rates of CINs (**D**) in the indicated groups; *P* < 0.01 by 1-way ANOVA, **P < 0.01 vs. water group by Tukey’s post hoc test; *n =* 49 neurons from 5 male and 2 female mice (Water), 31 neurons from 4 male and 2 female mice (Alcohol 1-d), and 36 neurons from 4 male mice (Alcohol 21-d). (**E**) Chronic alcohol did not change evoked CIN firing. Left and middle: Sample traces of membrane potentials generated in the indicated groups in response to a series of 500-ms current injections. Right: The input-output relationship between the injected current magnitude and the CIN firing frequency in water and alcohol groups; *P* > 0.05 by 2-way RM ANOVA; *n =* 16 neurons from 4 male mice (Water) and 13 neurons from 3 male mice (Alcohol).

Having shown that chronic alcohol intake reduced thalamic inputs onto DMS CINs, we asked whether alcohol also altered the spontaneous spiking of these tonically active neurons. We trained ChAT-eGFP mice to consume alcohol for 8 weeks using the intermittent-access 2-bottle choice drinking procedure. CINs were identified by their green fluorescence (Figure 3A), and spontaneous firing of DMS CINs was measured using cell-attached recording, 24 hours and 21 days after the last alcohol exposure (Figure 3B). We found that chronic alcohol consumption decreased the interspike interval (Figure 3C) and significantly increased the firing frequency over time (Figure 3D; *F*_(2,112)_ = 5.69, *P* < 0.01). In contrast, our measurement of intrinsic excitability using whole-cell recording did not find any significant difference in the evoked firing of DMS CINs from the water and alcohol groups (Figure 3E; *F*_(1,27)_ = 0.93, *P* > 0.05). These results suggest that chronic alcohol consumption increases the spontaneous activity of DMS CINs.

**Figure 4:**
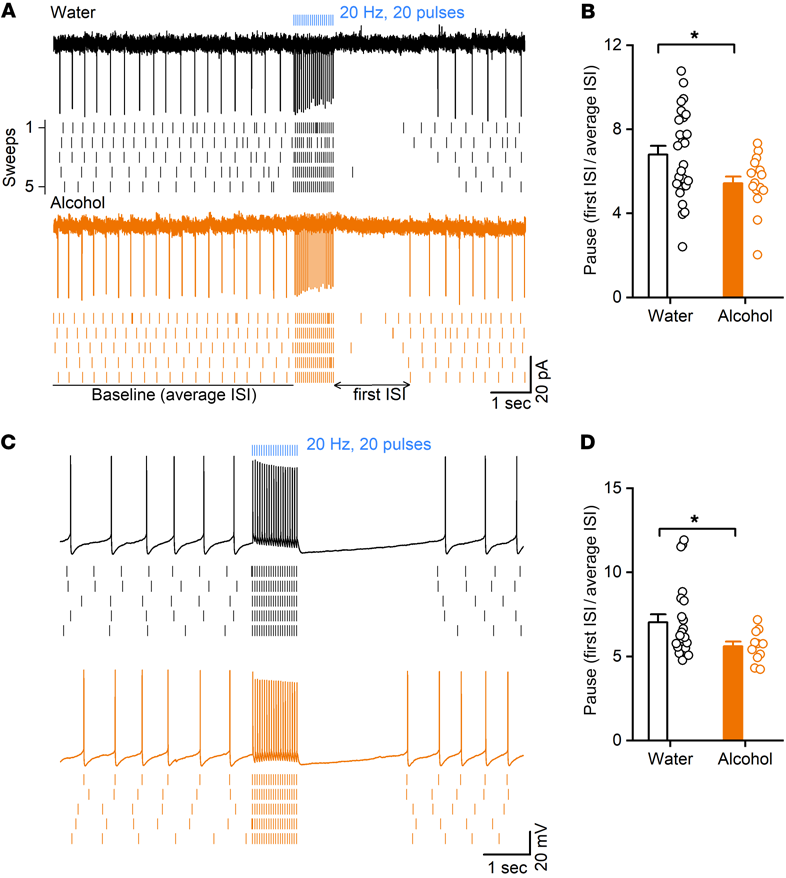
Chronic alcohol consumption shortens pause response of DMS CINs. ChAT-Cre Ai32 mice were trained to consume 20% alcohol for at least 8 weeks. Then DMS slices were prepared 24 hours after last alcohol exposure, and optically evoked burst-pause responses of CINs were measured. (**A**) Sample traces of burst-pause responses of a CIN from the water (top) and alcohol (bottom) groups using the cell-attached recording. ISI, interspike interval. (**B**) The pause durations in the indicated groups; *P < 0.05 by unpaired *t* test; *n =* 26 neurons from 3 male and 2 female mice (Water) and 16 neurons from 3 male mice and 1 female mouse (Alcohol). The pause duration is defined by the first ISI right after optical stimulation divided by baseline average ISI before the optical stimulation. (**C**) Sample traces of burst-pause responses of a CIN from the water (top) and alcohol (bottom) groups using whole-cell recording. (**D**) The pause durations in the indicated groups; *P < 0.05 by unpaired *t* test; *n =* 22 neurons from 3 male and 2 female mice (Water) and 11 neurons from 3 male mice (Alcohol).

CINs exhibit characteristic burst-pause firing, which is important for regulating MSN activity. Next, we investigated the effects of chronic alcohol intake on the burst-pause firing of CINs. To induce burst-pause response of CINs, we expressed ChR2 in CINs by crossing transgenic mice expressing Cre recombinase under the control of the choline acetyltransferase (ChAT) promoter (ChAT-Cre mice) with transgenic mice with Cre-dependent ChR2-eYFP expression (Ai32 mice) (37). ChAT-Cre Ai32 mice were trained to consume 20% alcohol for 8 weeks using the intermittent-access 2-bottle choice drinking procedure. Twenty-four hours after the last alcohol exposure, we prepared striatal slices and measured optically evoked burst-pause responses of DMS CINs. We found that the pause duration was significantly shorter in CINs from the alcohol group than in those from the water control group using cell-attached recording (Figure 4, A and B; *t*_(40)_ = 2.32, *P* < 0.05). We also observed similar results with whole-cell recording (Figure 4, C and D; *t*_(31)_ = 2.06, *P* < 0.05).

### Chronic alcohol consumption impairs CIN-induced suppression of NMDA receptor–mediated glutamatergic transmission in DMS D1-MSNs.

**Figure 5:**
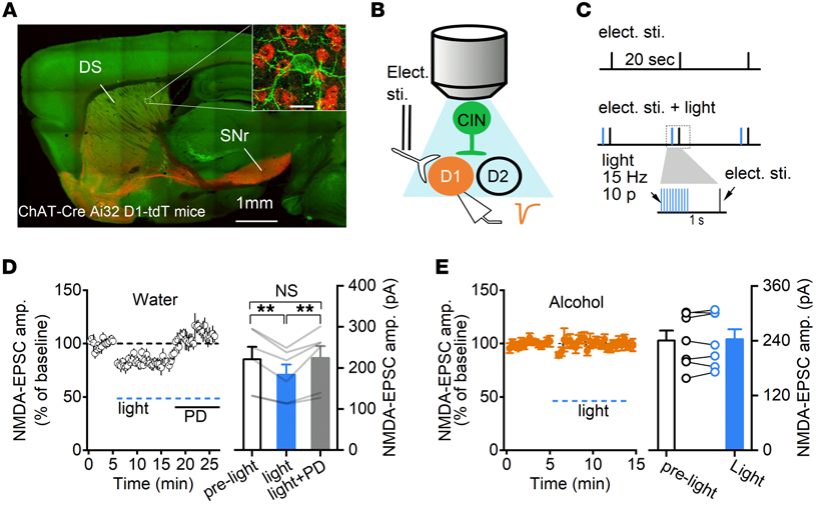
Chronic alcohol consumption impairs CIN-mediated suppression of glutamatergic transmission in DMS D1-MSNs. (**A**) Sample image of a sagittal section from 9 ChAT-Cre Ai32 D1-tdT mice. Inset shows a green CIN with several red D1-MSNs (scale bar: 20 μm). DS, dorsal striatum; SNr, substantia nigra pars reticulata. (**B**) Schematic of the electrical and optical stimulation and selective recording of D1-MSNs. The stimulating electrodes were placed in the DMS close to the recording pipette. (**C**) Schematic of the electrical and light stimulation protocols. Electrical stimulation (top) was delivered every 20 seconds, 1 second after the delivery of a burst of 473-nm light (2 ms of 10 pulses at 15 Hz) (middle and bottom). (**D**) The amplitude of NMDAR-mediated EPSCs before light stimulation, during light stimulation, and during light stimulation in the presence of the muscarinic M4 antagonist PD 102807 (PD; 1 μM) showed that optogenetic excitation of DMS CINs caused an M4 receptor–dependent suppression of NMDAR activity in D1-MSNs; *P* < 0.01 by 1-way RM ANOVA, **P < 0.01 vs. the light group by Tukey’s post hoc test; *n =* 7 neurons from 4 male mice and 1 female mouse per group. (**E**) Chronic alcohol consumption abolished CIN-induced suppression of NMDAR-EPSCs; *P* > 0.05 by paired *t* test; *n =* 7 neurons from 4 male mice per group.

CINs regulate flexible behaviors by modulating MSN activity. After characterizing the effects of chronic alcohol consumption on DMS CIN activity, we investigated how alcohol intake might affect CIN-mediated modulation of MSNs, leading to changes in striatal output. In striatal circuits, endogenous cholinergic signaling is known to modulate NMDA receptor–mediated (NMDAR-mediated) synaptic responses in D1-MSNs by acting on muscarinic M4 receptors (M4Rs) (26), which are downregulated in alcohol use disorder (44). We, therefore, examined whether endogenous acetylcholine release induced by optogenetic excitation of CINs altered NMDAR-EPSCs in DMS D1-MSNs. To achieve this, we generated triple-transgenic ChAT-Cre Ai32 D1-tdT mice, in which CINs expressed ChR2-eYFP and D1-MSNs contained tdT (Figure 5A). Stimulating electrodes were placed within the striatum to elicit glutamatergic transmission, and we patched the red D1-MSNs and excited CINs by delivering blue light through the objective lens (Figure 5B). After NMDAR-mediated EPSCs were recorded for 5 minutes (baseline), blue light (2 ms, 10 pulses at 15 Hz) was delivered 1 second prior to each electrical stimulation, and EPSCs were continuously monitored for 10 minutes (Figure 5C). We found that optogenetic excitation of CINs significantly reduced the NMDAR-EPSC amplitude in D1-MSNs. We further confirmed that this effect was mediated by muscarinic M4Rs, as subsequent application of an antagonist of this receptor, PD 102807 (1 μM) (45), completely abolished the CIN-mediated suppression of NMDAR-EPSCs (Figure 5D; *F*_(2,10)_ = 13.56, *P* < 0.01). We found that chronic alcohol consumption completely abolished this CIN excitation–induced suppression of NMDAR-EPSCs in D1-MSNs (Figure 5E; *t*_(6)_ = –0.68, *P* > 0.05). Taken together, these data indicated that excitation of DMS CINs activated muscarinic M4Rs to suppress NMDAR-EPSCs in DMS D1-MSNs and that chronic alcohol consumption attenuated this suppression.

### Chronic alcohol consumption compromises CIN-mediated short-term facilitation of glutamatergic transmission in DMS D2-MSNs.

**Figure 6:**
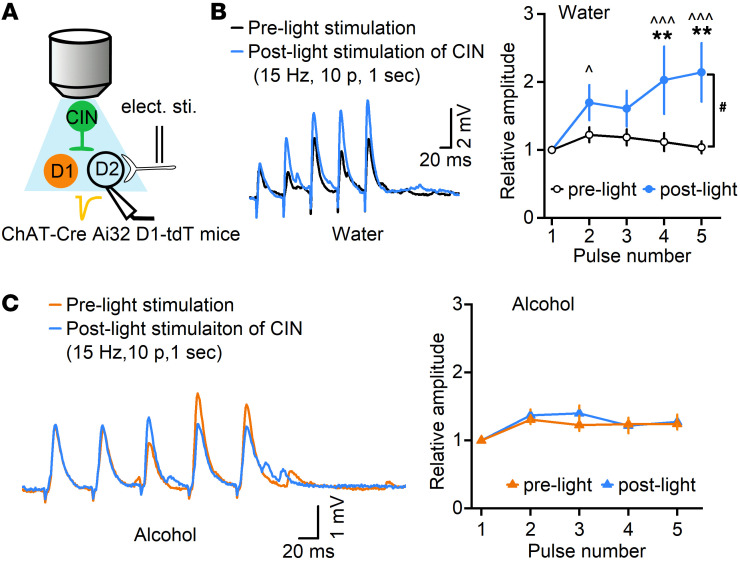
Chronic alcohol intake compromises CIN-mediated short-term facilitation of glutamatergic transmission in DMS D2-MSNs. (**A**) Schematic of electrical and light stimulation and whole-cell recording of D2-MSNs in ChAT-Cre Ai32 D1-tdT mice. Putative D2-MSNs were identified by their absence of fluorescence. (**B**) Left: Sample traces showing that electrical stimulation led to 5 EPSPs in D2-MSNs before and after light-mediated excitation of CINs. Electrical stimulation was delivered every 20 seconds, 1 second after the delivery of a burst of 473-nm light (2 ms of 10 pulses at 15 Hz). Right: Calculation of the relative amplitudes of 5 EPSPs detected short-term facilitation in water control mice after light-mediated excitation of CINs. EPSPs were normalized to the first one; ^#^*P* < 0.05 by 2-way RM ANOVA; **P < 0.01 vs. the same pulse number in the pre-light group by Tukey’s post hoc test; ^*P* < 0.05, ^^^*P* < 0.001 vs. pulse number 1 within the post-light group by Tukey’s post hoc test; *n =* 9 neurons from 5 male mice and 1 female mouse per group. (**C**) Left: Sample traces showing the EPSPs before and after light stimulation of CINs in the alcohol group. Right: Calculation of the relative amplitudes of EPSPs in the alcohol group did not identify any change after light stimulation of CINs; *P* > 0.05, 2-way RM ANOVA; *n =* 12 neurons from 4 male mice per group.

Having found that chronic alcohol consumption impaired CIN-mediated regulation of glutamatergic transmission in D1-MSNs, we next examined whether it altered CIN-mediated regulation of glutamatergic transmission in another major MSN type, the D2-MSN. We used ChAT-Cre Ai32 D1-tdT mice, in which putative D2-MSNs were identified as nonfluorescent (Figure 6A). Thalamic stimulation of cholinergic activity has been shown to cause short-term facilitation of AMPA receptor–mediated EPSPs (AMPAR-EPSPs) in D2-MSNs (17). We thus recorded electrically evoked AMPAR-EPSPs in D2-MSNs using the current-clamp recording. Five EPSPs were measured before and 1 second after light-mediated stimulation of CINs in mice that had been exposed to alcohol or water only. Compared with amplitudes recorded before light stimulation, we found that direct light stimulation (15 Hz, 10 pulses, 1 second before electrical stimulation) of CINs caused short-term facilitation of EPSP amplitudes in the water group (Figure 6B; *F*_(1,8)_ = 5.66, *P* < 0.05), as expected. Interestingly, there was also a main effect of pulse number (*F*_(4,32)_ = 3.89, *P* < 0.05), in that later electrical pulses generated higher relative EPSP amplitudes than earlier pulses (Figure 6B; vs. pulse 1: *q* = 4.53, *P* < 0.05 [pulse 2]; *q* = 6.67, *P* < 0.001 [pulse 4]; *q* = 7.41, *P* < 0.001 [pulse 5]). In contrast, light stimulation of CINs failed to potentiate the EPSP amplitudes in the alcohol group (Figure 6C; *F*_(1,11)_ = 0.91, *P* > 0.05). These results demonstrated that chronic alcohol consumption compromised CIN-mediated short-term facilitation of AMPAR-mediated transmission in DMS D2-MSNs.

### The alcohol-induced impairment of reversal learning is rescued by in vivo optogenetic potentiation of PfN-to-CIN transmission.

**Figure 7:**
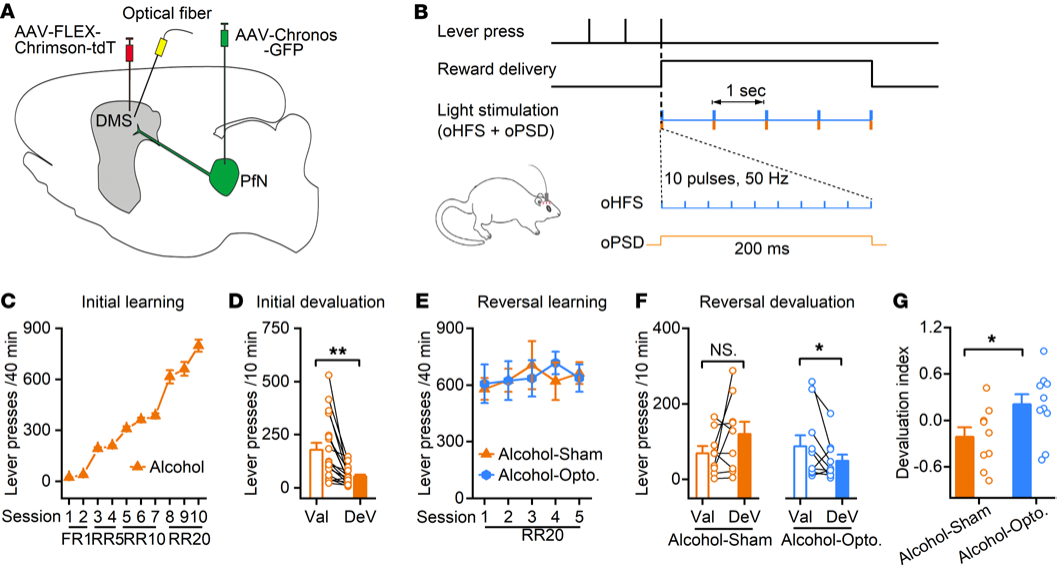
Optogenetic stimulation of PfN-to-CIN synapses in the DMS rescues the alcohol-induced impairment of reversal learning. (**A**) Schematic of viral injection and optical fiber implantation. ChAT-Cre rats were bilaterally infused with AAV-FLEX-Chrimson-tdT and AAV-Chronos-GFP into the DMS and PfN, respectively. Optical fibers were bilaterally implanted into the DMS. Rats were then trained using the same schedule as in Figure 1. (**B**) Optical stimulation protocol used during the reversal learning. Rats pressed the lever to receive a reward and light stimulation, which was time-locked to the reward delivery. Light stimulation contained 5 repeats of dual light stimulus within a 5-second reward delivery period. Each repeat consisted of optogenetic high-frequency stimulation (oHFS; 473 nm, 10 pulses, 50 Hz) and optogenetic postsynaptic depolarization (oPSD; 590 nm, 200 ms). (**C**) The initial acquisition learning curve. (**D**) Outcome-specific devaluation testing showed that rats pressed the DeV lever significantly fewer times than the Val lever; **P < 0.01 by paired *t* test. (**E**) There was no significant difference in lever pressing between the 2 groups during the reversed contingency training sessions; *P >* 0.05 by 2-way RM ANOVA. (**F**) Outcome-specific devaluation after reversed action-outcome contingency learning showed that the sham group still interacted more with the DeV lever (which was the Val lever during initial learning), while the group that received light stimulation showed successful devaluation after the reversed action-outcome contingency; NS, *P* > 0.05, and *P < 0.05 by paired *t* test. (**G**) The devaluation index was significantly higher in the opto group than in the sham group; *P < 0.05 by unpaired *t* test; *n =* 14 male and 5 female rats for **C** and **D**; *n =* 6 male and 3 female rats (Alcohol-sham) and 8 male and 2 female rats (Alcohol-opto) for **E**–**G**.

The above evidence points to the key roles of DMS CINs in mediating the detrimental effect of chronic alcohol intake on cognitive flexibility. Lastly, we aimed to alleviate this detrimental effect by manipulating the PfN-to-CIN connectivity. It has been shown that a global enhancement of the neuronal activity of CINs through pharmacogenetics failed to rescue the impairment of reversal learning in aged mice (10), indicating the need for a more targeted modulation of CINs by thalamostriatal processes. Therefore, we infused AAV-Chronos-GFP into the PfN and AAV-FLEX-Chrimson-tdT into the DMS of ChAT-Cre rats for selective manipulation of PfN-to-CIN synapses. Optical fibers were implanted into the DMS (Figure 7A). After recovery from surgery, rats were trained using the schedule described in Figure 1. Once the rats acquired the initial action-outcome contingencies (Figure 7, C and D), they were divided into 2 groups: the alcohol-opto group received time-locked light stimulation (Figure 7B) during the reversal learning; the alcohol-sham group underwent the same procedure as the alcohol-opto group except the lasers were not turned on. Both groups showed similar acquisition of initial action-outcome contingencies and initial devaluations (Supplemental Figure 5). During reversal training, we delivered optogenetic high-frequency stimulation of PfN inputs and “optogenetic postsynaptic depolarization of DMS CINs, a dual-channel optogenetic protocol that we recently developed to induce long-term potentiation in vivo (24). We found that there was no significant difference in terms of lever presses between the 2 groups (Figure 7E; *F*_(1,16)_= 0.002, *P* > 0.05). However, our analysis of the relative contributions of goal-directed versus habitual behavior following contingency reversal showed that the sham group pressed more devalued levers, indicating habitual behavior carrying over from initial learning; whereas the light stimulation group still favored the nondevalued lever, indicating new goal-directed behavior (Figure 7F; *t*_(8)_ = –1.52, *P* > 0.05 for sham group; *t*_(9)_ = 1.91, *P* < 0.05 for light stimulation group). The devaluation index was therefore significantly higher in light-stimulated rats as compared with their sham controls (Figure 7G; *t*_(17)_ = –2.23, *P* < 0.05). These results indicated that the alcohol-induced impairment of cognitive flexibility was restored by selective potentiation of thalamic inputs onto DMS CINs.

## Discussion

**Figure 8:**
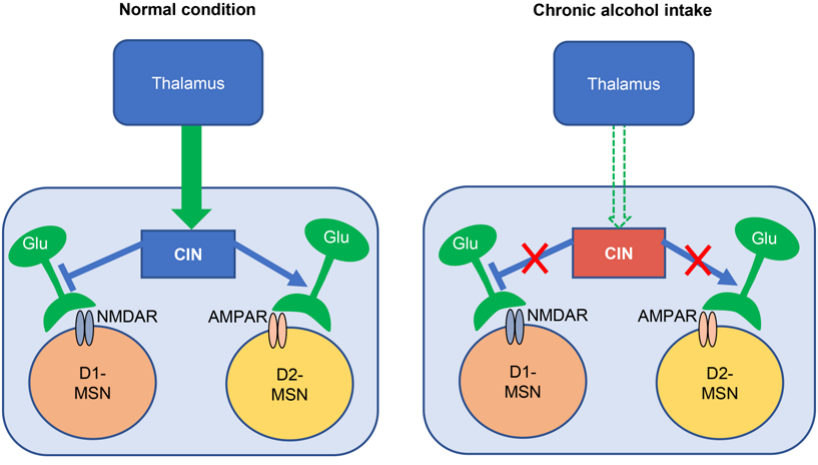
Schematic diagram showing the effects of chronic alcohol intake on the thalamic inputs onto CINs and their modulation of glutamatergic transmission to D1-MSNs and D2-MSNs in the striatum. Chronic alcohol consumption reduces thalamic excitatory inputs onto DMS CINs and increases their spontaneous firing, which makes them less prone to be modulated by external signals. In the meantime, the CIN-mediated inhibition of glutamatergic transmission in D1-MSNs and the CIN-mediated short-term facilitation of glutamatergic transmission in D2-MSNs are compromised after chronic alcohol intake, which could change striatal outputs and lead to behavioral inflexibility.

In this study, we demonstrated that chronic alcohol exposure and withdrawal reduced goal-directed cognitive flexibility and caused a long-lasting suppression of thalamostriatal inputs onto DMS CINs and a shortened pause response along with the increased spontaneous firing of these neurons. Furthermore, chronic alcohol consumption and withdrawal impaired CIN-mediated downregulation of glutamatergic transmission in D1-MSNs, as well as CIN-mediated short-term upregulation of glutamatergic transmission in D2-MSNs. Our data suggest that chronic alcohol consumption compromises the thalamostriatal regulation of glutamatergic transmission in MSNs via CINs (Figure 8), providing insight into how chronic alcohol consumption changes from casual, flexible drinking to compulsive intake.

In individuals with alcohol use disorder, a progressive loss of cognitive flexibility eventually results in compulsive alcohol-drinking behavior. Growing evidence suggests that the dorsal striatum is a key hub in the regulation of cognitive flexibility (6, 7). We found that chronic alcohol consumption impaired the reversal of action-outcome contingency, indicating behavioral inflexibility. It is highly likely that this behavioral change is due to effects on the dorsal striatum (46, 47). Within this brain region, CINs play a critical role in regulating reversal learning (9, 10), which is essential in the reversal phase but not in the initial memory acquisition (9) — a fact that highlights the importance of CIN activity for new state formation, or the revision of behavior to accommodate a new situation (9, 48). Given that the present study found that chronic alcohol consumption and withdrawal affected glutamatergic transmission from the thalamus to striatal CINs, it is highly possible that this disruption induces a deficit in goal-directed action selection (9). This prediction was supported by our devaluation results, which indicated that chronic alcohol intake and withdrawal impaired devaluation during contingency reversal but did not impair contingency acquisition. This impairment includes reduced lever presses and failed devaluation tests during and after reversal learning, respectively. Since animals use both goal-directed and habitual strategies in operant conditioning (1, 49), the devaluation failure suggests that alcohol-treated animals only use a habitual strategy to press levers under new action-outcome contingencies, leading to reduced presses. Interestingly, a change in lever pressing is not required for cognitive flexibility impairment (9). We found that while optogenetic strengthening of PfN-to-CIN transmission did not alter lever presses during reversal learning, it enabled alcohol-treated animals to use a goal-directed strategy, resulting in the successful devaluation of new action-outcome contingencies. Taken together, these findings suggest that chronic alcohol intake impairs the flexibility of striatum-mediated goal-directed behavior.

Having observed these behavioral effects of alcohol, we next investigated whether the thalamic-to-CINs circuit is affected. Most previous in vitro and in vivo studies have demonstrated that thalamic stimulation produces burst-pause activity in CINs, which modulates D1- and D2-MSNs (17, 25). Therefore, thalamic glutamatergic transmission to CINs is a key component of this circuit (17). By selectively activating thalamic inputs to the DMS, the present study demonstrates that thalamic input modulates CIN activity and thus controls the striatal MSN network. Furthermore, we found that chronic alcohol consumption decreased thalamic glutamatergic transmission to CINs. This effect lasts 21 days after the last alcohol consumption, suggesting that alcohol induces long-lasting synaptic depression of thalamostriatal inputs onto DMS CINs. Since our behavioral experiments were conducted within 21 days after the last alcohol exposure, the long-lasting alcohol-induced reduction of thalamic inputs onto CINs is likely to contribute to the corresponding cognitive flexibility deficits. Our study of paired-pulse ratios found that the probability of glutamate release did not decrease, indicating that this effect was not mediated by a decrease in thalamic activity (Figure 2). The mechanisms underlying this alcohol-induced reduction of thalamic inputs onto DMS CINs need further investigation.

Our experiments involving direct optogenetic stimulation of CINs showed that activation of CINs exerted a complex and powerful influence on specific types of striatal outputs. The burst stimulation of CINs at 15 Hz, which is close to the burst firing frequency observed under physiological conditions (17), suppressed NMDAR-mediated glutamatergic inputs onto D1-MSNs and facilitated AMPAR-mediated glutamatergic transmission in D2-MSNs. The depression of D1-MSNs and facilitation of D2-MSNs by our direct optical activation of CINs were consistent with previous studies that employed electrical stimulation of the thalamus (17). The integrated effect on DMS MSNs, namely a decrease in the D1-direct pathway output and an increase in the D2-indirect pathway output, is to activate the striatopallidal network to suppress action (“no go”). Our results showed that chronic alcohol intake disrupted CIN-mediated depression of D1-MSNs and facilitation of D2-MSNs. These results are consistent with acute alcohol impairing the ability of thalamostriatal inputs to modulate a subsequent corticostriatal glutamatergic response in MSNs (3). The loss of CIN-mediated action suppression promotes “go” action. A previous study also found that glutamatergic transmission increased in D1-MSNs after alcohol consumption (23). The effect of this disruption, which increases the relative activity of D1-MSNs and reduces that of D2-MSNs, is to reduce action suppression and make a “go” outcome more likely.

The exact mechanisms of alcohol-induced loss of CIN-mediated modulation of MSNs are unclear. CINs are known to corelease acetylcholine and glutamate (50). A recent study reports that a loss of acetylcholine release from CINs promotes habitual behaviors and reduces cognitive flexibility, whereas silencing glutamate release from CINs favors the development of goal-directed behaviors (51). Therefore, CIN-released acetylcholine and glutamate have opposite roles in regulating cognitive flexibility where acetylcholine promotes the formation of new association and glutamate suppresses it. We found that chronic alcohol intake persistently reduced thalamic inputs to CINs, which is likely to suppress burst cholinergic activity and acetylcholine release, thereby contributing to alcohol-induced cognitive inflexibility. Interestingly, we discovered that chronic alcohol intake enhanced the spontaneous firing of CINs, which may also elevate tonic glutamate release in the striatum. Since silencing glutamate release from CINs enables the learning of flexible goal-directed behaviors (51), this purported elevation in tonic glutamate release from CINs is expected to reduce cognitive flexibility. Cholinergic muscarinic M4Rs, functionally coupled with the NMDAR, are only expressed in D1-MSNs (52). A previous study reports that M4Rs negatively regulate D1-MSN function (53). Consistently, we observed that optogenetic CIN stimulation inhibited NMDAR-EPSCs in D1-MSNs in an M4R-dependent manner, suggesting that endogenous acetylcholine activates M4Rs to suppress the EPSCs. Importantly, this suppression was lost in alcohol-drinking animals, but the mechanism remains unknown. A recent study reveals that M4R levels in D1-MSNs are reduced after alcohol intake (54). This reduced M4R expression is likely to cause little M4R activation in response to optogenetic CIN stimulation in alcohol-treated animals. Consequently, we did not detect any CIN stimulation–induced suppression of NMDAR-EPSCs.

Interestingly, the present study used a chronic alcohol consumption procedure, and we found an increase in the spontaneous firing of CINs, in contrast to the inhibiting effect of acute alcohol on CIN firing (3). It is not uncommon that chronic and acute drugs have opposite effects due to the adaptation response of the organism. For example, acute morphine administration increased the spontaneous firing of dopamine neurons in the ventral tegmental area (VTA) (55, 56), while chronic morphine administration and withdrawal greatly reduced the spontaneous activity of VTA dopamine neurons (57, 58). We also found that acute alcohol suppressed NMDA activity while chronic alcohol consumption enhanced NMDA function (59). As for the function of CINs, it may not directly correlate with the baseline activity; in other words, increased baseline firing does not mean enhanced function of CINs. It has been shown that aged mice have increased spontaneous firing of CINs and exhibit impairments in reversal learning of action-outcome contingencies (10). Pharmacogenetic direct stimulation of CINs in the DMS did not alleviate the impairment of reversal learning in aged mice (10). It seems that the extent to which CINs can be modulated plays a more important role in their function than does their baseline firing. With increased baseline firing, CINs could be less prone to be modulated, such as the shortened pause response observed in our study (Figure 4). By selectively strengthening the thalamic inputs onto DMS CINs, we were able to rescue the impairment of reversal learning in rats with a history of chronic alcohol consumption (Figure 7). While rats consume less alcohol than mice (5–6 vs. 15–20 g/kg per 24 hours; refs. 23, 28, 33, 34, 60–62), the blood ethanol concentrations are equivalent in these models (80–160 mg/dL), as mice metabolize ethanol more quickly than rats (33, 60, 63, 64). A limitation of this study is that behavioral experiments were performed in rats and electrophysiological recordings were conducted in mice. Rats perform better than mice in operant learning, and we have previously conducted optogenetic manipulation of rat operant self-administration (24, 32, 65). However, diverse transgenic mouse lines allow us to perform cell type–specific stimulation and recordings. Importantly, previous studies have shown that alcohol intake reduces cognitive flexibility in mice (66–68). While our ChAT-Cre mice were generated using the IRES knockin technique and express the same levels of vesicular acetylcholine transporters (VAChT) and ChAT proteins as the wild type (69), the BAC-transgenic ChAT-Cre rats and ChAT-GFP mice overexpress VAChT (70) and exhibit hypercholinergic tone (71). This hypercholinergic tone unlikely contributes to the alcohol-induced loss of cholinergic modulation of cognitive flexibility in the present study.

CINs are a major source of acetylcholine within the striatum, and their dense terminals primarily synapse with MSNs. We generated triple-transgenic mice to induce selective optogenetic excitation of CINs and allow fluorescent identification of D1-MSNs. We found that direct optogenetic excitation of CINs elicited a stimulation-evoked firing response followed by a pause (Figure 4). The burst-pause firing of CINs is intricately linked with dopamine activity in the striatum (72). Indeed, the pause duration was reduced by blocking of dopamine D2 receptors (Supplemental Figure 4). This result is consistent with the finding that the thalamically evoked pause is dependent on dopamine release and D2 receptor activation (17). Previous in vivo studies show that CINs exhibit burst-pause responses to salient cues (73, 74). The reversal of action-outcome contingencies serves as salient stimulation to trigger burst-pause responses in CINs. The burst firing of CINs increases the release of acetylcholine, which stops ongoing actions by inhibiting D1-MSNs (17). During the pause period of CIN firing, dopamine neurons usually increase their firing and elevate striatal dopamine levels (72). This dopamine elevation facilitates plasticity in D1-MSNs (75, 76), which likely strengthens the formation of new action-outcome contingencies. The alcohol-induced reduction of pause durations is expected to limit the learning of new action-outcome contingencies, and thus reduce cognitive flexibility.

In summary, DMS CINs modulate striatal circuits via burst-pause firing, which is triggered by inputs from the thalamus. Alcohol consumption disrupts this modulation by reducing thalamic excitation of DMS CINs and increasing their spontaneous activity. Our research demonstrates that alcohol attenuates both CIN-mediated inhibition of glutamatergic transmission in D1-MSNs and CIN-mediated short-term facilitation of glutamatergic transmission in D2-MSNs. These effects have the potential to impair cognitive flexibility by preventing the formation of new goal-directed behaviors. Our findings provide a base of evidence for the development of new therapeutic strategies to enhance cognitive flexibility in patients with alcohol use disorder.

## Methods

### Animals

ChAT-eGFP (stock 007902), ChAT-Cre (stock 031661), VGluT2-Cre (stock 016963), Drd1a-tdTomato (D1-tdT, stock 016204), and Ai32 (stock 012569) mice were purchased from The Jackson Laboratory. All mice were backcrossed onto a C57BL/6 background. VGluT2-Cre or ChAT-Cre mice were crossed with Ai32 to generate VGluT2-Cre Ai32 or ChAT-Cre Ai32 lines. VGluT2-Cre (or ChAT-Cre) and ChAT-eGFP (or D1-tdT) mice were crossed with Ai32 to generate VGluT2-Cre Ai32 ChAT-eGFP or ChAT-Cre Ai32 D1-tdT triple-transgenic mice. The alcohol drinking levels among these lines were indistinguishable (21.8 ± 0.7 g/kg per 24 hours). Both male and female mice were used for electrophysiology studies. Male Long-Evans rats (3 months old) purchased from Harlan Laboratories were used for behavioral testing. Long-Evans-Tg(ChAT-Cre) rats were purchased from Rat Resource & Research Center (stock 00658). ChAT-Cre rats were bred in-house. Both male and female ChAT-Cre rats (2 months old) were used for behavioral testing. The drinking levels did not differ between ChAT-Cre and wild-type Long-Evans rats (6.9 ± 0.6 g/kg per 24 hours). Animals were housed individually at 23°C under a 12-hour light/12-hour dark cycle, with lights on at 7:00 am. Food and water were provided ad libitum.

### Reagents

PD 102807 (catalog 1671) and DNQX (6,7-dinitroquinoxaline-2,3-dione; catalog 0189) were purchased from Tocris. LY367385 (catalog L4420), sulpiride (catalog S8010), picrotoxin (catalog P1675), and other reagents were obtained from MilliporeSigma.

### Intermittent-access 20% alcohol 2-bottle choice drinking procedure

This procedure was conducted as described previously (23, 24, 31–34, 77, 78). Briefly, animals were given concurrent access to 1 bottle of alcohol (20%, in water) and 1 bottle of water for 24-hour periods, which were separated by 24- or 48-hour periods of alcohol deprivation. Alcohol intake (g/kg/d) was calculated by determining the weight of 20% alcohol solution consumed and multiplying this by 0.2. Water control animals only had access to water.

### Operant conditioning training

Operant conditioning was conducted in rats after the 2-bottle choice drinking procedure. During conditioning training, rats no longer had access to alcohol. Blinding was applied to behavioral experiments. An independent observer coded and randomized animals using a computer-generated blinding algorithm. Researchers in the laboratory trained rats without knowing the treatment plan for the animals. Food was restricted to maintain 80% of the original body weight of the animals for the duration of behavioral studies.

#### Magazine training.

This procedure was adapted from Bradfield and Balleine (35). After 5 days of food restriction, rats were trained for magazine entries for 20 minutes on 2 consecutive days. During these training sessions, a reinforcer (either a food pellet or 0.1 mL sucrose solution) was delivered along with illumination of the magazine light for 1 second with a random interval between each reinforcer (on average 60 seconds). The house light was illuminated throughout the session, and no levers were available during magazine training. An equal number of rats received either 20 food pellets or 20 sucrose deliveries during the first training session and were then switched to receive the other reward in the second training session.

#### Acquisition of initial contingencies.

Following magazine training, rats were trained to access different reinforcers via lever pressing over the next 10 days. Each session consisted of 4 blocks (2 blocks per lever), separated by a 2.5-minute time-out during which no levers were available, and all lights were extinguished. Only 1 lever was available during each block (pseudorandom presentation), which lasted for 10 minutes or until 10 reinforcers had been earned. For half of the animals in each group, the left lever was associated with food pellet delivery and the right lever with sucrose solution delivery. The remaining animals were trained using the opposite pairs of action-outcome contingencies. Lever training started with a fixed ratio 1 (FR1) schedule in which every lever press resulted in the delivery of a reinforcer. After 2 days of FR1 training, the training schedule was elevated to a random ratio 5 (RR5) schedule for the next 3 days, during which a reinforcer was delivered after an average of 5 lever presses. An RR10 training schedule was then used for 3 days, followed by an RR20 schedule for the final 2 days.

#### Devaluation test.

After the final RR20 training, devaluation testing was performed for 2 days. On both days, rats were habituated in a dark, quiet room (different from the operant room) for 30 minutes, then were given ad libitum access to either the food pellets (25 g placed in a bowl) or the sucrose solution (100 mL in a drinking bottle) in a devaluation cage for 1 hour. The devaluation cage was similar to their home cage but with new bedding. The rats were then placed in the operant chamber for a 10-minute extinction choice test. Both levers were extended during this test, but no outcomes were delivered in response to any lever press. On the second devaluation day, the rats were pre-fed, as described, with the other reward before repeating the same extinction test. If rats fail to perform during the devaluation test, then the pre-feed reward amount and duration need to be tailored to individual animals. Lever presses (LP) were recorded, and those on the lever that the rat had learned to associate with the nondevalued reward were termed LP_valued_, while those on the lever associated with the devalued reward were termed LP_devalued_. The devaluation index [(LP_valued_ – LP_devalued_)/(LP_valued_ + LP_devalued_)] was then used to determine the extent of goal-directed versus habitual behavior.

#### Contingency reversal and devaluation testing.

After the devaluation test, rats were retrained on their current action-outcome contingencies for 1 day. The contingencies were then reversed so that the lever that previously delivered food now delivered sucrose, and the rats were trained using the RR20 schedule. All other procedures were unchanged. The contingency reversal training lasted for 4–5 days. The rats then underwent devaluation testing again using the procedure described above.

### Electrophysiology

Slice electrophysiology was performed as previously described (24, 62, 77, 79). Animals were weaned around postnatal day 21 and consumed 20% alcohol for 6–8 weeks in the intermittent-access 2-bottle choice drinking procedure. Animals were sacrificed 24 hours or 21 days after their last alcohol consumption, and 250-μm coronal sections containing the striatum were prepared in an ice-cold cutting solution containing (in mM): 40 NaCl, 148.5 sucrose, 4 KCl, 1.25 NaH_2_PO_4_, 25 NaHCO_3_, 0.5 CaCl_2_, 7 MgCl_2_, 10 glucose, 1 sodium ascorbate, 3 sodium pyruvate, and 3 myoinositol, saturated with 95% O_2_ and 5% CO_2_. Slices were then incubated in a 1:1 mixture of cutting solution and external solution at 32°C for 45 minutes. The external solution contained the following (in mM): 125 NaCl, 4.5 KCl, 2.5 CaCl_2_, 1.3 MgCl_2_, 1.25 NaH_2_PO_4_, 25 NaHCO_3_, 15 sucrose, and 15 glucose, saturated with 95% O_2_ and 5% CO_2_. Slices were then maintained in external solution at room temperature until use.

Slices were perfused with the external solution at a flow rate of 3–4 mL/min at 32°C. The CINs and MSNs in the DMS were identified either by differential interference contrast or by fluorescence. Whole-cell patch-clamp and cell-attached recordings were made using a MultiClamp 700B amplifier controlled by pClamp 10.4 software (Molecular Devices). For cell-attached and whole-cell current-clamp recordings, we used a K^+^-based intracellular solution containing (in mM): 123 potassium gluconate, 10 HEPES, 0.2 EGTA, 8 NaCl, 2 MgATP, 0.3 NaGTP (pH 7.3), with an osmolarity of 270–280 mOsm. For whole-cell voltage-clamp recordings, we used a Cs-based solution, containing (in mM): 119 CsMeSO_4_, 8 tetraethylammonium chloride, 15 HEPES, 0.6 EGTA, 0.3 Na_3_GTP, 4 MgATP, 5 QX-314 chloride, 7 phosphocreatine. The pH was adjusted to 7.3 with CsOH.

For measurement of spontaneous CIN firing, cell-attached recordings were conducted in the voltage-clamp mode. In whole-cell current-clamp recordings, evoked action potentials were elicited by 500-ms stepped current injections at 30-pA increments from –120 pA to +120 pA. Optogenetically evoked CIN firing was induced by light stimulation (473 nm, 2 ms, 15 Hz, 10 pulses) through the objective lens. Bipolar stimulating electrodes were positioned 100–150 μm away from the recording electrode that was used to record glutamatergic transmission in MSNs. To measure NMDAR-EPSCs, the neurons were recorded in the presence of DNQX and with magnesium-free external solution. All of the measurements were conducted in the presence of the GABA_A_ receptor antagonist picrotoxin (100 μM). The experiments in Figure 5 were conducted in the presence of the mGluR1/5 antagonist LY367385 (10 μM).

### Stereotaxic surgery and histology

The rabies helper viruses (AAV8-DIO-RG and AAV8-DIO-TVA-mCherry), AAV-Chrimson-tdT, AAV-FLEX-Chrimson-tdT, and AAV-Chronos-GFP were purchased from the University of North Carolina Vector Core. The pseudotyped rabies viruses, EnvA-SADΔG-mCherry and EnvA-SADΔG-GFP (2.04 × 10^8^ transduction units per mL), were obtained from the Salk Institute Vector Core.

Stereotaxic viral infusions were performed as described previously (23, 24, 28, 62). Briefly, mice were anesthetized using isoflurane and mounted in a rodent stereotaxic frame (Kopf Instruments). The skin was opened to uncover the skull and expose bregma and lambda, and the location of the desired injection site. A 3-axis micromanipulator was used to measure the spatial coordinates for bregma and lambda. Small drill holes were made in the skull at the appropriate coordinates, according to the Paxinos atlas (80). Two microinjectors were loaded with 0.5 μL of a 1:1 mixture of AAV8-DIO-RG and AAV8-DIO-TVA-mCherry, and then lowered into the posterior DMS (anteroposterior [AP], 0.0 mm; mediolateral [ML], ±1.87 mm; dorsoventral [DV], –2.90 mm). This helper virus mixture was infused into the brain at a rate of 0.1 μL/min. To avoid backflow of the virus, microinjectors were left in place for 10 minutes after the infusion was complete. After their removal, the skin was sutured, and the mice were allowed to recover for 3 weeks prior to the infusion of pseudotyped rabies virus (EnvA-SADΔG-mCherry or EnvA-SADΔG-eGFP). The rabies virus was injected at the same site and using the same injection volume as the initial helper virus injection. To prevent coincident rabies infection along the injection tract, the rabies virus was infused into adapted coordinates (AP, 0.0 mm; ML, ±2.42 mm; DV, –2.94 mm) at an angle of 10° (81) to the previous injection. The modified coordinates were calculated by measuring from the midline and parallel to the dorsal-ventral axis. The coordinates for mouse PfN injection (AAV-Chrimson-tdT, 0.5 μL) were AP, –2.2 mm; ML, ±0.7 mm; DV, –3.5 mm. Those for ChAT-Cre rat DMS (AAV-FLEX-Chrimson-tdT) were AP, 0.0 mm; ML, ±2.8 mm; DV, –4.85 mm. Those for ChAT-Cre rat PfN (AAV-Chronos-GFP) were AP, –4.2 mm; ML, ±1.25 mm; DV, –6.2 mm. For rats, 1 μL to 1.2 μL of the virus was infused in each hemisphere. After virus injections, optical fibers (300-μm core fiber secured to a 1.25-cm ceramic ferrule with 5 mm of fiber extending past the end of the ferrule) were bilaterally implanted into the DMS right on the top of virus injection sites; coordinates were AP, 0.0 mm; ML, ±2.8 mm; DV, –4.8 mm. Implants were secured on the skull using metal screws and dental cement (Henry Schein) and covered with denture acrylic (Lang Dental). The incision was closed around the head cap and the skin was adhered with Vetbond to the head cap. Rats were monitored for 1 week or until they resumed normal activity.

The histology procedure was performed as described previously (24, 62, 82). Briefly, mice were anesthetized and perfused intracardially with 4% paraformaldehyde in PBS. Whole brains were taken out and placed into 4% paraformaldehyde in PBS for postfixation overnight (4°C), then moved to 30% sucrose in PBS (4°C) and allowed to sink to the bottom of the container before preparation for sectioning. Frozen brains were cut into 50-μm coronal sections on a cryostat. A confocal laser-scanning microscope (Fluorview-1200, Olympus) was used to image these sections with a 470-nm laser (to excite eYFP and GFP) and a 593-nm laser (to excite tdT). All images were processed using Imaris 8.3.1 (Bitplane).

### Statistics

All data are expressed as the mean ± SEM. Statistical significance was assessed using the unpaired or paired *t* test or 2-way repeated-measures ANOVA followed by Tukey’s test for post hoc comparisons. Statistical significance was set at *P* less than 0.05.

### Study approval

All animal care and experimental procedures were approved by Texas A&M University’s Institutional Animal Care and Use Committee (protocol 2019-0285) and were conducted in accordance with the National Research Council *Guide for the Care and Use of Laboratory Animals* (National Academies Press, 2011).

## Author contributions

JW conceived, designed, and supervised all the experiments in the study. TM wrote the first draft of the manuscript, and JW, TM, ZH, YC, LNS, RJS, and YZ revised the manuscript. ZH, TM, and XZ designed and performed electrophysiology experiments and analyzed the data. ZH, XX, and MC designed and performed the behavior experiments and analyzed the data. HG and XW conducted histology experiments. TM and ZH contributed equally to this research as co–first authors. The order of co–first authors was determined by the temporal order of research contribution and agreed upon by the co–first authors. Co–first authors have the right to list themselves first for purposes of their curriculum vitae and biosketch.

## Supplementary Material

Supplemental data

## Version 1 12/23/2021

In-Press Preview

## Version 2 02/15/2022

Electronic publication
